# CpG stimulation of chronic lymphocytic leukemia cells induces a polarized cell shape and promotes migration *in vitro* and *in vivo*

**DOI:** 10.1371/journal.pone.0228674

**Published:** 2020-02-10

**Authors:** Maria Dampmann, André Görgens, Michael Möllmann, Florian Murke, Ulrich Dührsen, Bernd Giebel, Jan Dürig

**Affiliations:** 1 Department of Hematology, University Hospital, University of Duisburg-Essen, Essen, Germany; 2 Department of Laboratory Medicine, Karolinska Institutet, Stockholm, Sweden; 3 Institute for Transfusion Medicine, University Hospital Essen, University Duisburg-Essen, Essen, Germany; 4 Department of Internal Medicine, St. Josef Hospital, Essen, Germany; European Institute of Oncology, ITALY

## Abstract

In order to accomplish their physiological functions leukocytes have the capability to migrate. As a prerequisite they need to adopt a polarized cell shape, forming a leading edge at the front and a uropod at rear pole. In this study we explored the capability of chronic lymphocytic leukaemia (CLL) cells to adopt this leukocyte-specific migration phenotype. Furthermore, we studied the impact of the Toll-like receptor 9 (TLR9) agonists CpGs type A, B and C and the antagonist oligodesoxynucleotide (ODN) INH-18 on the cell polarization and migration process of primary human CLL cells. Upon cultivation, a portion of purified CLL cells adopted polarized cell shapes spontaneously (range 10–38%). Stimulation with CpG ODNs type B (ODN 2006) and CpGs type C (ODN 2395) significantly increased the frequency of morphologically polarized CLL cells, while ODN INH-18 was hardly able to act antagonistically. Like in human hematopoietic stem and progenitor cells, in morphologically polarized CLL cells CXCR4 was redistributed to the leading edge and CD50 to the uropod. Coupled to the increased frequencies of morphologically polarized cells, CpGs type B and C stimulated CLL cells showed higher migration activities *in vitro* and following intravenous injection higher homing frequencies to the bone marrow of immunocompromised NOD.Cg-*Prkdc*^*scid*^ *Il2rg*^*tm1Wjl*^/SzJ (NSG) mice. Thus, presumably independent of TLR-9 signaling, CpGs type B and C promote the cellular polarization process of CLL cells and their ability to migrate in *vitro* and *in vivo*.

## Introduction

Chronic lymphocytic leukemia (CLL) is the most common leukemia in the western hemisphere. Although many patients show a rather indolent course of the disease, about one third of the patients suffer from an aggressive course that is only insufficiently controlled by immunochemotherapy. Therefore, there is a constant aim to develop and test new drugs in translational and clinical studies.

Overexpression of the oncogene bcl-2 is known to be a key player in causing defects in apoptosis leading to leukemic expansion of affected B cells. Furthermore, bcl-2 participates in mediating chemotherapy resistance and worsens the clinical outcome [1]. Hence, it has been selected as a target for anti-CLL therapy. Already in 1999 bcl-2 antisense oligonucleotides had been introduced as novel drugs for CLL treatment [2]. One of the most encouraging compounds at this time was oblimersen/G3139, which showed beneficial effects in a multicentric Phase I to II clinical trial [3] as well as in combination with fludarabine and cyclophosphamide in a randomized Phase III clinical study [4]. Despite the positive effects of oblimersen, these studies did not investigate whether oblimersen affects the survival of CLL cells directly by the down-regulation of its target gene bcl-2 or indirectly by exerting immune stimulatory effects. In 2001, Jahrsdörfer and colleagues reported CG dinucleotides contained in oblimersen exert the same anti-CLL effects than CpG oligonucleotides, which induce the expression of costimulatory and antigen-presenting molecules on normal as well as malignant B-cells [5]. Thus, it appears more likely that oblimersen does not specifically affect the activity of bcl-2, but like CpG motifs of bacterial DNA activates the mammalian immune system via toll-like receptor 9 signaling (TLR9) [6].

This notion is further supported by results of a translational study, in which we compared the novel antisense bcl-2 inhibitor SPC2996 with oblimersen. SPC2996 was designed to have a higher affinity to bcl-2, to contain a better biostability and to mediate less immunostimulatory impacts than oblimersen. As expected, SPC2996 led to a rapid clearance of leukemic cells and affected the expression of 466 identified genes differentially than oblimersen. However, expression analyses did not reveal a significant effect on the expression of bcl-2 [7]. In contrast, gene enrichment analysis of the genes being induced by SPC2996 treatment identified multiple genes participating in the toll-like receptor signaling pathway [7]. Coupled to this, serum levels of tumor necrosis factor-α (TNF-α) and macrophage inflammatory protein-1α (MIP-1α), cytokines being regulated by TLR signaling [8, 9], were significantly increased in CLL patients following SPC2996 treatment [7]. Zent and colleagues applied CpG oligonucleotides in a Phase I clinical trial and observed the same rapid reduction of peripheral blood lymphocytes of treated patients as we did in our Phase I trial [7, 10]. This led us to hypothesize that CpG motifs rather promote B-cell stimulation than affecting the expression level of bcl2. Due to the fact that upon applying National Cancer Institute (NCI) criteria no durable clinical responses were measurable [7, 10], it appears less likely that the CpG oligonucleotides induce apoptosis of B- and CLL cells. Thus, we assumed that CpG motifs stimulate B- and CLL cells to exit from the circulation and invade into tissues, a process known as extravasation. Extravasation depends on migratory capabilities of extravasating cells [11]. Previously, we have investigated migratory capabilities of normal human hematopoietic stem and progenitor cells (HSPCs), in more detail. We demonstrated that as a prerequisite for their migration, HSPCs need to adopt a polarized morphology, forming a leading edge at the front and a uropod at the rear pole [12, 13]. Any drugs affecting the establishment of the morphologically recognizable cell polarity were shown to also affect the HSPCs’ *in vitro* migratory capabilities as well as their capability to home and engraft into the bone marrow of immune deficient mice [13, 14]. As HSPCs and the different immune cell types including CLL cells uniquely adopt amoeboid migration phenotypes in mammals, which can also be analyzed at the molecular level, e.g. by the redistribution of intercellular adhesion molecules and chemokines [12, 13, 15–17], we decided to investigate the impact of CpG oligonucleotides on the cellular polarization and migration process of CLL cells in more detail.

Dependent on their exact sequence different types of CpG oligonucleotides were described which i) mainly stimulate IFNγ production in dendritic cells (DCs, CpGs type A), ii) mainly activate B cells (CpGs type B), and iii) stimulate IFNγ production in DCs and also activate B cells (CpGs type C) [18, 19]. Thus, to be able to identify differences of the different CpGs types on the cell polarization and migration behavior of CLL cells, we included a representative of each CpG type in our study.

## Material and methods

### Cell culture experiments

Peripheral blood was obtained from CLL patients after written informed consent according to our institutional guidelines. The study was approved by the Ethics Commission of the University of Duisburg-Essen (reference 14-6080-BO). Patient characteristics are listed in S1 Table. Peripheral blood mononuclear cells (PBMCs) were isolated employing Lymphoprep (STEMCELL Technologies, Köln, Germany) density-gradient centrifugation. Afterwards CLL cells were further purified by CD3+ depletion (EasySep ^®^ Human CD3 Positive Selection Kit, STEMCELL Technologies), resulting in a purity of CD19+/CD5+ cells of >95% as determined by flow cytometry. CLL cells (1.5 x 106 cells/ml) were taken into culture in serum-free medium (EX-CELL^®^ 610-HSF Serum-Free Medium, Sigma-Aldrich, Taufkirchen, Germany). CpG oligonucleotides type A (ODN 2216), type B (ODN 2006), type C (ODN 2395) (all InvivoGen, San Diego, USA) or H2O control were added at a concentration of 0.25 μM (app. 2 μg/ml). For analysis of TLR-9 inhibition, TLR-9 inhibitory ODN INH-18 (InvivoGen, San Diego, USA) was added at concentration of 0.25 μM, 0.5 μM, 1 μM, 2.5 μM or 5 μM together with 0.25 μM CpG type B (ODN 2006). After 48 hours of incubation in an 5% CO2 and at 37°C atmosphere cells were observed by time-lapse microscopy. Thereafter, the cell numbers and viability were assessed by trypan blue staining (Sigma-Aldrich). Cell culture supernatants were frozen at -80° C until usage. All experiments were performed in duplicates.

### Analysis of cell polarization and cell migration by time-lapse microscopy

Cell polarization and motility was monitored by time-lapse video-microscopy recorded with a Zeiss Axio Observer.Z1 microscope equipped with a motorized xyz stage (Ludl Electronics, Ltd., Hawthorne, USA). Treated and control CLL cells were kept in a climate chamber (37°C and 5% CO_2_ with humidity) and analyzed simultaneously in duplicates with the mark/find function of the microscope. Images in a time-course of 3h were taken every 110 seconds, a full representative time-lapse video is shown in S1 Movie. Cellular polarization was analyzed by manually marking all cells on the first picture as being morphologically polarized or non-polarized. Each comma shaped like-cell, the cells with protrusions, were marked as a polarized cell; spherical cells were counted as non-polarized cells. The marks were counted by the Cell Counter plugin of ImageJ 1.43u (NIH, Bethesda, USA) (S2A Fig). The motility of the cells was quantified by manual tracking of 10 morphologically polarized and 10 non-polarized individual cells for all 100 images using ImageJ 1.43u with the Manual Tracking Plugin (Fabrice Cordelières) (S2B Fig). The Chemotaxis and Migration Tool 2.0 (ibidi, Martinsried, Germany) was used for the centering correction, plotting of tracking data and calculation of mean velocity and distance (S2C Fig).

### Quantification of MIP-1α and TNF-α in cell culture supernatants by enzyme-linked immunosorbant assay (ELISA)

MIP-1α (CCL3) and TNF-α concentration was measured in cell culture supernatant of CpG stimulated CLL cells using Quantikine Kits, according to the manufacturer’s instructions (R&D Systems, Wiesbaden, Germany). The absorbance was recorded by a MRX microplate reader and analyzed by Revelation software version 4.22 (Dynatech Laboratories, Denkendorf, Germany). Intra- and inter-plate assay precision determined by coefficients of variance (CV) were 4.9% and 7.6% for TNF-α assay and 2.0% and 5.1% for MIP-1α assay, as reported by the manufacturer.

### Quantification of cell polarity by imaging flow cytometry

For imaging flow cytometry (IFCM) experiments CLL cells were freshly isolated and cultured in serum-free medium for 24 hours in presence or absence of 0.25 μM CpGs type B (ODN 2006) as described above. Cells were fixed with 0.4% Paraformaldehyd (PFA) for 5 min, washed in PBS, and stained for 20 min at 4°C in the dark with conjugated antibodies. The following directly conjugated mouse anti-human antibodies were used: CD19-PC5 (clone J3119, Beckmann-Coulter, Krefeld, Germany), CXCR4-PE (clone 2B11/CXCR4; BD Pharmingen, San Jose, USA), CD50-FITC (clone TU41; BD Pharmingen). To identify dead cells DAPI (4,6-diamidino-2-phenylindole; 200 ng/mL; Roche, Mannheim, Germany) was added to before measuring cells on AMNIS Image Stream X (Luminex, Austin, USA).

For the detailed investigation of morphologically round cells 20 intrinsically apolar cells and 20 intrinsically polarized cells were manually marked. Using the FindTheBestFeature tool of the AMNIS Image Stream IDEAS (Luminex) software allowing the most convincing discrimination of both types of morphologically round cells were identified. The ‘Homogeneity’ feature was identified being most suitable. Consequently, it was used to quantify the portion of intrinsically polarized cells in the presence or absence of CpG stimulation (S1 Fig).

### *In vivo* studies

NOD.Cg-*Prkdc*^*scid*^ *Il2rg*^*tm1Wjl*^/SzJ (NSG) xenograft studies were performed as described previously [20] with minor modifications. Animals purchased from Jackson Laboratory (Scaramento, California, USA) were bred and housed at the University Hospital Essen animal care facility. Animal experiments were performed in accordance with institutional guidelines approved by the Animal Care Committee of the University Duisburg-Essen (reference: G 1124/10-AZ: 87–51.04.2010.A133). Briefly, NSG mice of both genders (n = 12 per experiment, 3 experiments) were bred and handled under sterile conditions. Water was autoclaved and food was irradiated to ensure sterile conditions for the mice. PBMCs from CLL patients were freshly isolated as described above and stimulated with 5 μM CpGs type A, B, C or H_2_O *in vitro* for 2 hours in RPMI medium (Sigma-Aldrich). Mice were irradiated sublethally (3.5 Gy, approximately 30 min) before each a total of 5x10^7^ cells was injected into their tail veins. Experiments were performed in quadruples. For the harvest of human cells, mice were sacrificed by cervical dislocation after applying isoflurane anaesthesia 4 hours post transplantation. Bone marrow cells were harvested by flushing femoral and tibial bones with RPMI. Afterwards the cells were filtered through 100 μm nylon sieves (Becton Dickinson, Heidelberg, Germany). For engraftment analyses within the murine spleens the organ was mechanically homogenized, pressed through 100-μm nylon sieves (Becton Dickinson), and suspended in PBS. Erythrocytes were lysed using Pharmlyse reagent (Becton Dickinson) according to the manufacturer’s protocol. After resuspension in FACS Buffer (PBS supplemented with 2% FCS and 0.02% sodium azide) cells were stained with a cocktail of four different antibodies [CD5-FITC (clone C17F12, Becton Dickinson), CD19-PC5 (clone J3119, Beckmann Coulter), CD-38 PE (clone HB-7, Becton Dickinson) and CD45-APC (clone 2D1, Becton Dickinson)] for 30 min on. After washing, FACS buffer containing DAPI was added and flow cytometric analyses were performed on a LSR II flow cytometer (Becton Dickinson).

### Statistical analysis

For the statistical comparison of the cell culture and *in vivo* experiments two-sided Student’s t-test for paired samples was employed. For comparing ELISA results Mann-Whitney-U test was employed. Statistical analyses were performed with GraphPad Prism version 6 (GraphPad Software, San Diego, USA).

## Results

### CpG stimulation induces TNF-α and MIP-1α secretion and cellular polarization of CLL cells

**Fig 1:**
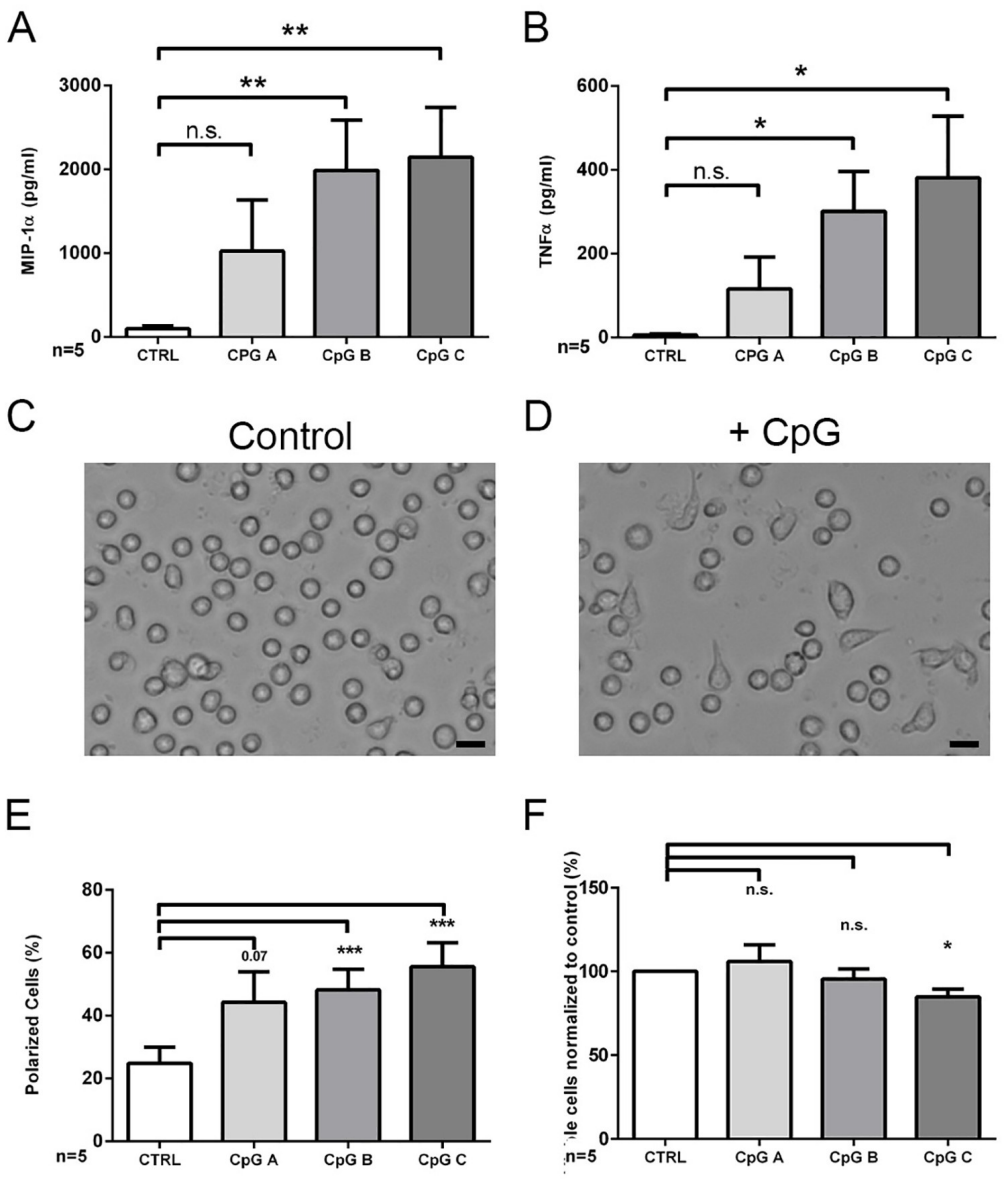
CpGs type B and C induce a polarized phenotype of CLL cells. A, B: Cytokines TNF-α (A) and MIP-1α in cell culture supernatant of CLL cells after 48 hours after cultivation without and with stimulation by 0.25 μM CpGs type A, B or C (CpG A, CpG B, CpG C; n = 5). Bars represent mean ± SEM. C, D: Brightfield images (20x objective; scale bar 10 μm) of CLL cells cultivated for 48 hours in serum-free medium in absence (A) or presence of CpGs type B (B). E, F: Cell polarization (E) and viability (F) of CLL cells assessed by manual counting 48 hours after cultivation without and with stimulation by 0.25 μM CpGs type A, B or C (n = 5). Bars represent mean ± SEM.

Freshly isolated CLL cells were cultured for 48h in the presence or absence of CpGs type A, B or C respectively, all at a concentration of 0.25 μM. To investigate whether TLR signaling was successfully activated, we determined the concentration of TNF-α and MIP-1α within respective supernatants. Upon stimulation with CpGs type B and C significantly higher concentrations of both cytokines were detected than in supernatants of CLL cells either being stimulated with CpGs type A or left unstimulated (Fig 1A and 1B). Related to the strength of cytokine secretion, CpG stimulation affected the cell morphology of cultured CLL cells. In five independent experiments, all performed in duplicates, significantly higher frequencies of morphologically polarized CLL cells were detected in cultures supplemented with 0.25 μM CpGs type B or C than in corresponding control cultures not being supplemented with any CpG molecules (Fig 1C and 1D). Notably, cell viability was not affected by the presence of CpGs types A and B and only slightly reduced in the presence of CpGs type C (Fig 1E and 1F). Titration experiments revealed that already at concentration of 0.125 μM CpG type B ODNs promote cell polarization of primary CLL cells and positively affect the cells’ viability (S3 Fig). Thus, in good association with the increased secretion of the proinflammatory cytokines following CpGs type B and C stimulation a higher proportion of CLL cells adopted a morphologically polarized cell phenotype.

### The TLR9 inhibitor ODN INH-18 only slightly reduces the cell polarization promoting activity of CpG type B

**Fig 2:**
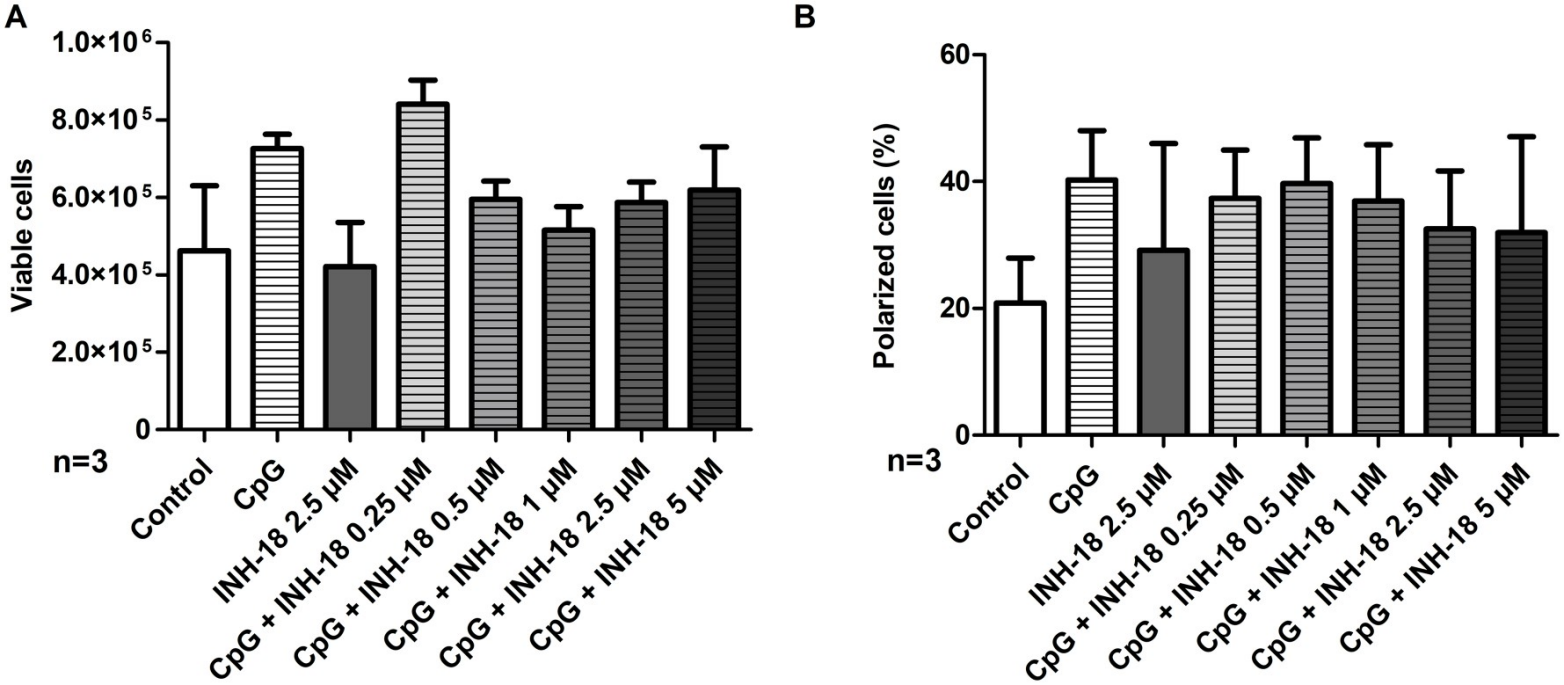
TLR9 antagonist ODN INH-18 hardly affects the cell polarization process of CpG type B stimulated primary CLL cells. Freshly isolated CLL cells were cultured in the presence of 0.25 μM of CpG type B and escalating doses of the TLR9 antagonist ODN INH-18 (0.25 μM–5 μM) (n = 3). Cell viability (A) and cell polarization (B) were assessed after 48h hours.

Next, we aimed to investigate whether CpG type B promotes cell polarization of primary CLL cells via TLR-9 signaling. To this end we decided to perform studies with the TLR-9 inhibitor ODN INH-18. At first we investigated the impact of different concentrations of ODN INH-18 (0.25 μM, 0.5 μM, 1.0 μM, 2.5 μM or 5 μM) on the cell viability of CpG type B stimulated primary CLL cells (n = 3). Upon analyzing 48 h post seeding a markedly decline of viable CLL cells were already observed at concentrations of 0.5 μM ODN INH-18 (Fig 2A). However, we did not observe any impact of lower doses of ODN INH-18 on the cell polarity promoting activity of CpG type B. A slight but not statistically significant reduction of polarized CLL cells was recorded when ODN INH-18 was added at a concentration of 2.5 or 5 μM (Fig 2B). Thus, our data show that the TLR-9 inhibitor ODN INH-18 effectively reduces cell viability but hardly affect the cell polarity promoting effect of CpG type B in CLL cells. Thus, our data suggest the cell polarity induction in CLL cells does not essentially depend on TLR-9 signaling.

### Morphologically polarized CLL mimic the cell polarity cues of morphologically polarized leukocytes and of hematopoietic stem and progenitor cells

**Fig 3:**
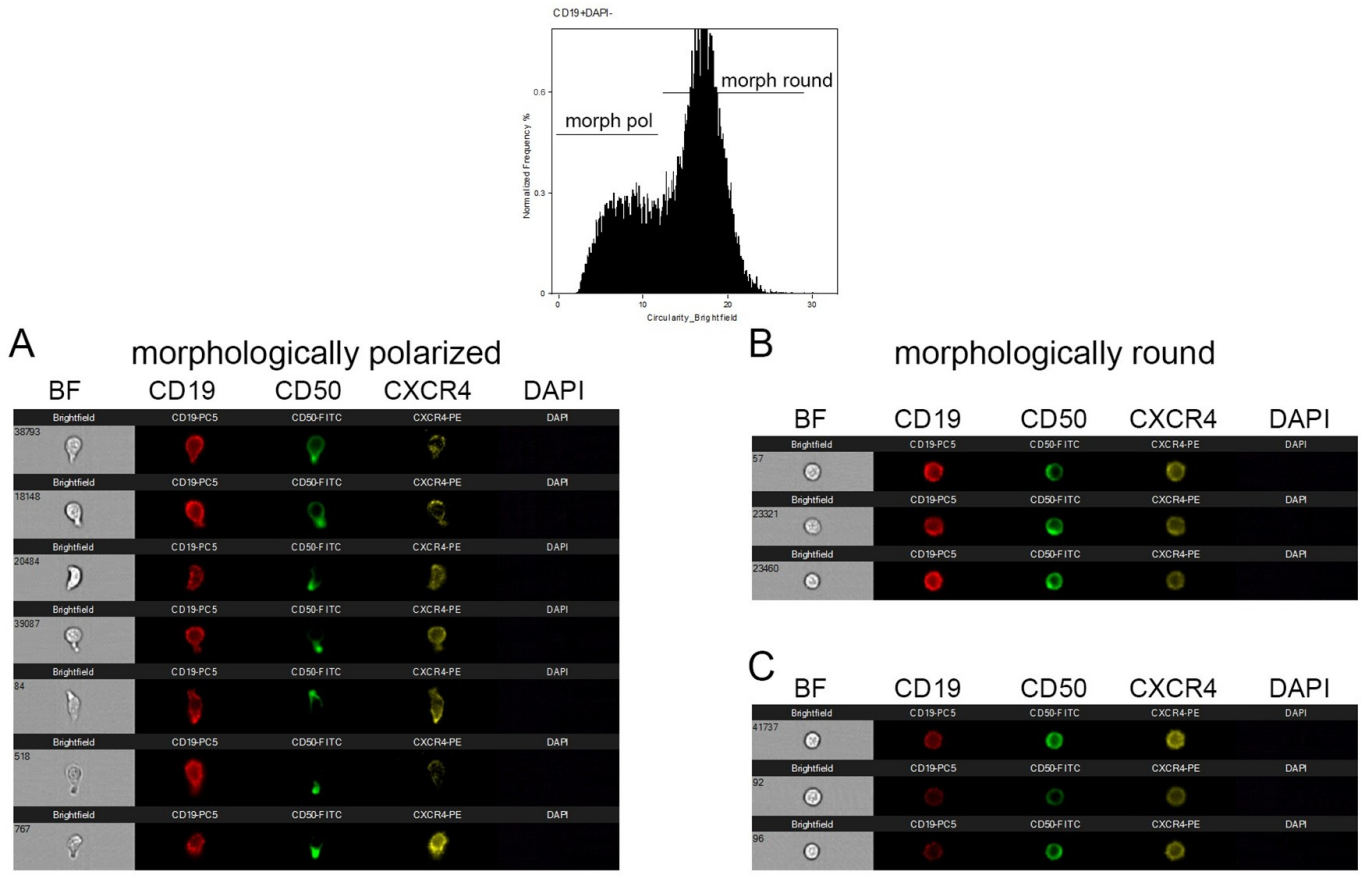
CLL cells polarize *in vitro* and redistribute selected cell surface antigens. CLL cells measured by IFCM. Cells were preincubated in serum-free medium for 24 hours, and stained with CD 19, CD50, CXCR4 and DAPI. In terms of cell polarity CLL cells were divided into three categories: i) cells with a spherical appearance and a diffuse subcellular distribution of CD50 and CXCR4 (B), ii) CLL cells with a spherical appearance and a polarized distribution of CD50 and CXCR4 (C) and iii) morphologically polarized CLL cells with CD50 being enriched at the rear and CXCR4 at the front (A).

**Fig 4:**
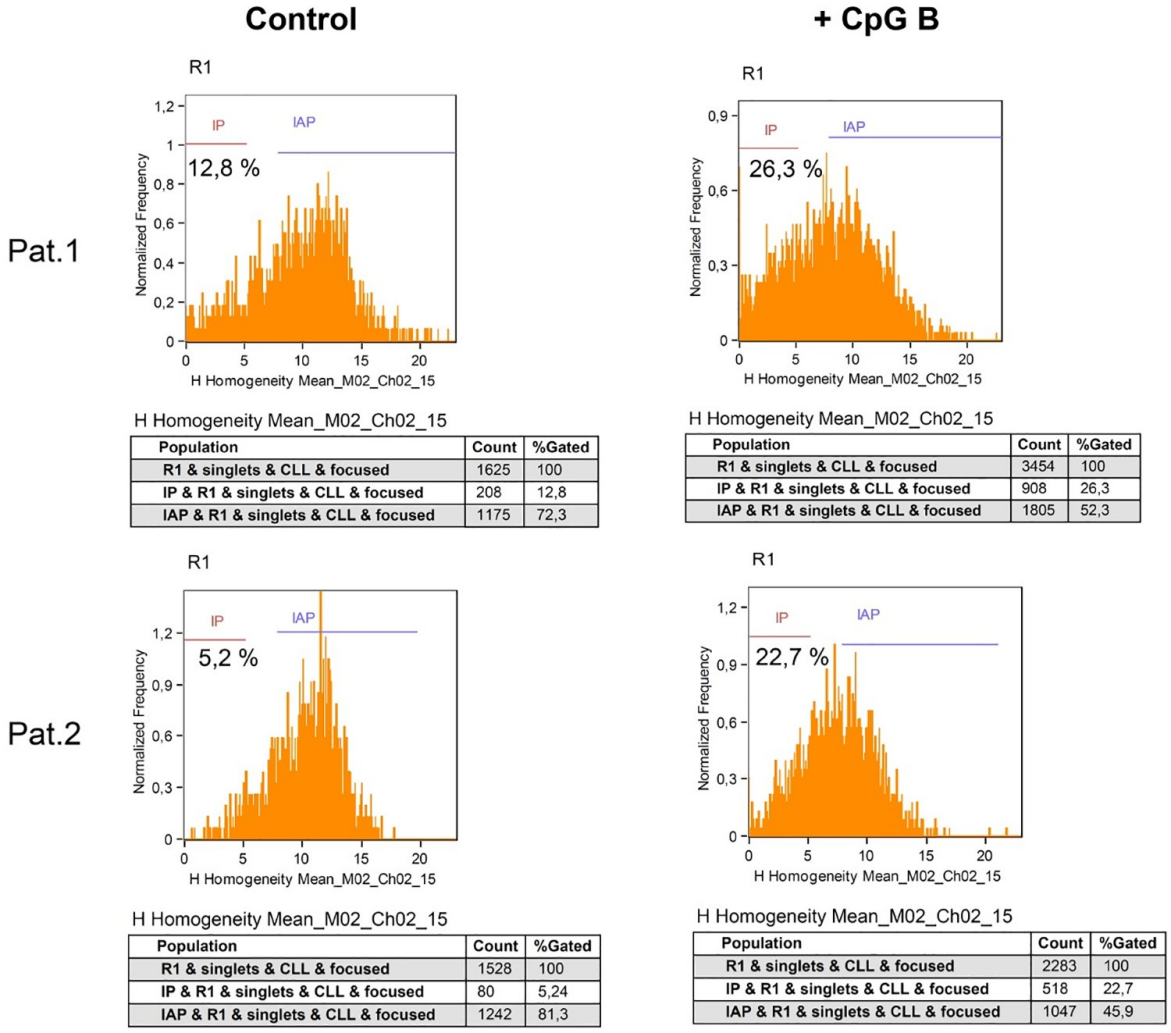
CpGs type B induce intrinsic polarization. CLL cells were incubated with or without CpGs type B (CpG B; 0.25 μM) for 24 h and subsequently analyzed by IFCM (Fig 2). The homogeneity feature was employed to best discriminate intrinsic apolar (IAP) cells from intrinsic polarized (IP) cells. The gating strategy is depicted in S1 Fig. The addition of CpGs type B to CLL cells increases intrinsic polarization.

Upon morphological polarization several leukocytes including HSPCs redistribute a number of antigens to either their front or rear pole, the leading edge or the uropod, respectively [12, 15, 16, 21, 22]. To investigate, whether CLL cells adopt the leukocyte/HSPC specific cell polarity phenotype, we studied the subcellular localization of CD50 and CXCR4, which we used as uropod and leading edge markers, respectively, in CpGs type B stimulated CLL cells exemplarily. After staining, CLL cells that had either been cultured in the presence or absence of CpGs type B were analyzed by imaging flow cytometry on the AMNIS ImageStream X platform. Comparable as in our previous study on HSPCs [13], three different cell polarization states of CLL cells could be discriminated (Fig 3): i) cells with a spherical appearance and a diffuse subcellular distribution of CD50 and CXCR4 (Fig 3B), ii) CLL cells with a spherical appearance and a polarized distribution of CD50 and CXCR4 (Fig 3C) and iii) CLL cells with CD50 being enriched at the rear and CXCR4 at the front (Fig 3A). Thus, like in other leukocytes, we found CD50 highly enriched in the uropod and CXCR4 in the leading edge of the morphologically polarized CLL cells. Upon discriminating the three different categories in control cultures lacking CpG molecules, most cells (72.3% patient 1 / 81.3% patient 2) revealed phenotypes of category i), 12.8% (patient 1) / 5.2% (patient 2) of category ii) and 14.9% / 13.5% of category iii). The relation of the categories shifted significantly, when CpG type B molecules were present within the cultures. Upon adding CpGs type B, 52.3% / 45.9% of the CLL cells fall in category i) 26.3% / 22.7% in category ii) and 21.4% / 31.4% in category iii) (Fig 4). Overall our data indicate that stimulated CLL cells adopt the leukocyte/HSPC specific cell polarity phenotype.

### CpGs type B and C promote morphological polarization but do not promote the motility of morphologically polarized cells

**Fig 5:**
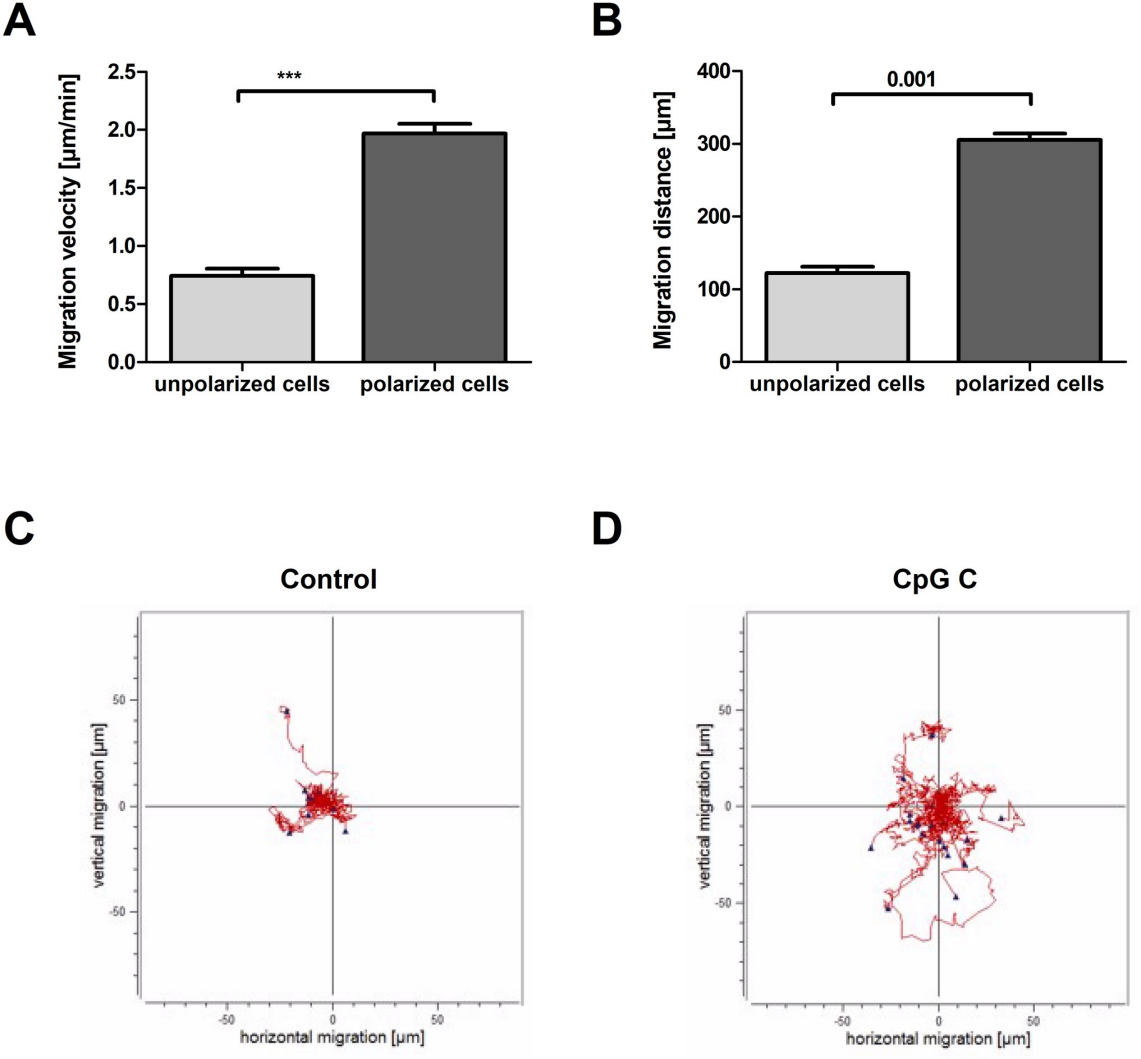
Morphologically polarized CLL cells have higher motilities than morphologically round cells. A, B: Migrated velocity (A) and migration distance (B) of morphologically round and polarized cells. The bar graphs show the mean migration velocity (A) and distance (B) of all conditions (with and without CpGs type A, B, or C [CpG A, CpG B, CpG C]). Bars represent mean ± SEM. C, D: Center-corrected migration plots of 10 polarized and 10 unpolarized cells in absence (C) or presence (D) of CpGs type C.

Previously, we demonstrated that morphological cell polarization is a prerequisite for human HSPCs to migrate *in vitro* and *in vivo* [13, 23]. Consequently, we investigated whether the frequencies of cultured CLL cells showing morphologically polarized phenotypes correlate with their *in vitro* migration capabilities. Upon recording cells by time-lapse microscopy and using the Chemotaxis and Migration tool for quantification (S2 Fig), we confirmed that morphologically polarized CLL cells migrate significantly faster and pass longer distances than morphologically round CLL cells (Fig 5). Comparing the motility of the morphologically polarized cells observed under the different culture conditions, we did not detect any significant differences. Thus, the presence of CpGs type B and C promoted the morphological cell polarization process, but had no recognizable impact on the motility of cells once being morphologically polarized.

### CpGs type B and C promote homing of CLL cells to the bone marrow of NSG mice

**Fig 6:**
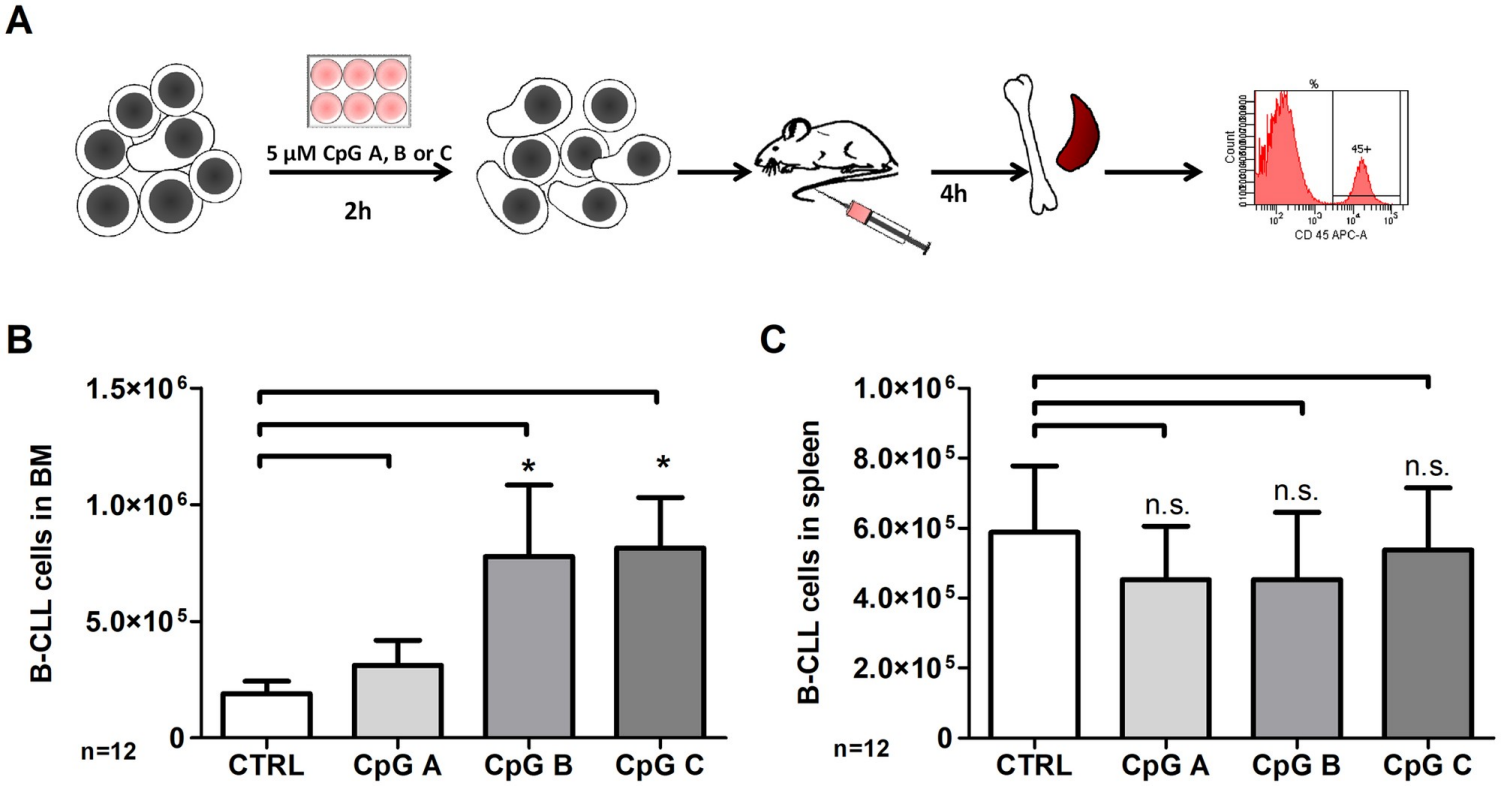
CpGs type B and C enhance CLL cell homing *in vivo*. CLL cells were preincubated *in vitro* with 5 μM NaCl, CpGs type A, B or C for 2 hours and subsequently injected in NSG mice (A). Homing of human CLL cells to the bone marrow (BM) (B) and spleen (C) was assessed after 4 hours by flow cytometry. Bars represent mean ± SEM.

To test the biological significance of our findings for their migration *in vivo*, i.e. their tissue homing capabilities, we transplanted CLL cells cultured for two hours in the presence or absence of CpG molecules into NSG mice and studied their presence in the spleen and in the bone marrow four hours after transplantation. Correlating with the percentage of morphologically polarized cells within the CLL cultures, more CpGs type B and C stimulated CLL cells homed to the bone marrow than CLL cells which were cultured in the presence of CpGs type A or in the absence of any CpGs (Fig 6). In contrast, no differences in the number of spleen invading cells were recognized. Thus, our data demonstrate that CpGs type B and C not only promote the cell polarization and migration of CLL cells *in vitro*, but also their capability to home to the bone marrow of transplanted mice.

## Discussion

Here we report that, *in vitro*, some CLL cells adopt polarized cell shapes spontaneously. The frequency of morphologically polarized cells is strongly increased upon addition of specific CpGs, apparently in a TLR-9 independent manner. Once being morphologically polarized CLL cells showed increased motilities *in vitro* and also homed in higher frequencies to the bone marrow of transplanted mice. As morphologically polarized CLL cells reflected the same motility *in vitro* independent of whether CLL cells were treated with CpGs or were left untreated, CpGs apparently do not affect the migration process itself.

Notably, we found significant differences between the different CpG types in their ability to induce morphologic cell polarization in CLL cells, while CpGs type B and C promoted cell polarization significantly, CpGs type A had only marginal impacts, which is related to the different structures and functions of these CpGs [24].

Compatible with the results of our study and as mentioned before, CpGs type A (e.g. ODN 2116) show only little B-cell activation [18], which is reflected by the relative low TNF-α and MIP-1α production of CpGs type A treated CLL cells. In contrast, as indicated by the increased TNF-α and MIP-1α production CpGs type B (e.g. ODN 2006) strongly activate B cells [19]. CpGs type C (e.g. ODN 2395) combine chemical features of CpGs type A and B [18], accordingly they also effectively stimulated the TNF-α and MIP-1α production. Since CpG type B and C but not type A treatment of CLL cells promoted the cell polarization of CLL cells and their migratory capabilities including their homing abilities, cellular polarization occurs, as in other leukocytes and HSPCs, in CLL cells as a consequence of their activation.

In this context it is worth mentioning that at least a proportion of CLL cells residing in the bone marrow survive current CLL therapies and become responsible for relapses of the disease [25, 26]. Thus, any drug which promotes cell polarization and homing of CLL cells into the bone marrow should be questioned as drugs being effective long-term. Indeed, as already mentioned in the introduction, therapeutic agents containing CpG motifs similar to CpG type B like oblimersen and SPC2996 failed to show effectivity in the clinical setting [3, 4, 7].

At the example of human HSPCs we have previously demonstrated that any condition affecting the establishment of a polarized cell shape attenuates the cells ability to migrate *in vitro* and to home and engraft into the bone marrow of immunocompromised mice following transplantation into their tail vein [13, 14]. Accordingly, novel drugs being able to block the cell polarization processes and/or homing of CLL cells specifically, might help—potentially in combination with current drugs—to significantly reduce relapse rates in CLL therapy in the future.

## Supporting information

S1 Fig Gating strategy to best discriminate intrinsic cell polarization at the molecular level. CLL cells were analyzed by IFCM as described in Fig 2. For morphologically round cells with polar (A) and diffuse (B) distribution of CD50 the Homogeneity Feature combined with the channel mask of channel 2 (M02) was applied to discriminate both cell populations in quantitative manners (C, D).
(TIF)

S2 Fig Assessment of polarization and migration of CLL cells. A: The fraction of polarized cells was determined by manual counting in ImageJ Green markers in the given example represent morphologically polarized cells, blue markers represent unpolarized cells. B: One single cell was tracked on 100 pictures of time-lapse microscopy and the track was analyzed by a Manual tracking plugin for ImageJ. C: Migration tracks of 10 polarized cells depicted by Chemotaxis and Migration tool.
(TIF)

S3 Fig Titration of CpG type B effects. CLL cells were incubated with rising concentration of CpG type B. Cell viability (A) and cell polarization (B) were assessed after 48h hours.
(TIF)

S1 Movie Time-lapse microscopy of CLL cells. Time-lapse microscopy of CLL cells in serum-free medium. The videos show 100 photos in 110 sec time intervals.
(AVI)

S1 Table (DOCX)
