# Supplementary figures and images for: CpG stimulation of chronic lymphocytic leukemia cells induces a polarized cell shape and promotes migration in vitro and in vivo
Source: PLoS One. 2020 Feb 10;15(2):e0228674. doi: 10.1371/journal.pone.0228674 (PMC7010256; doi:10.1371/journal.pone.0228674)

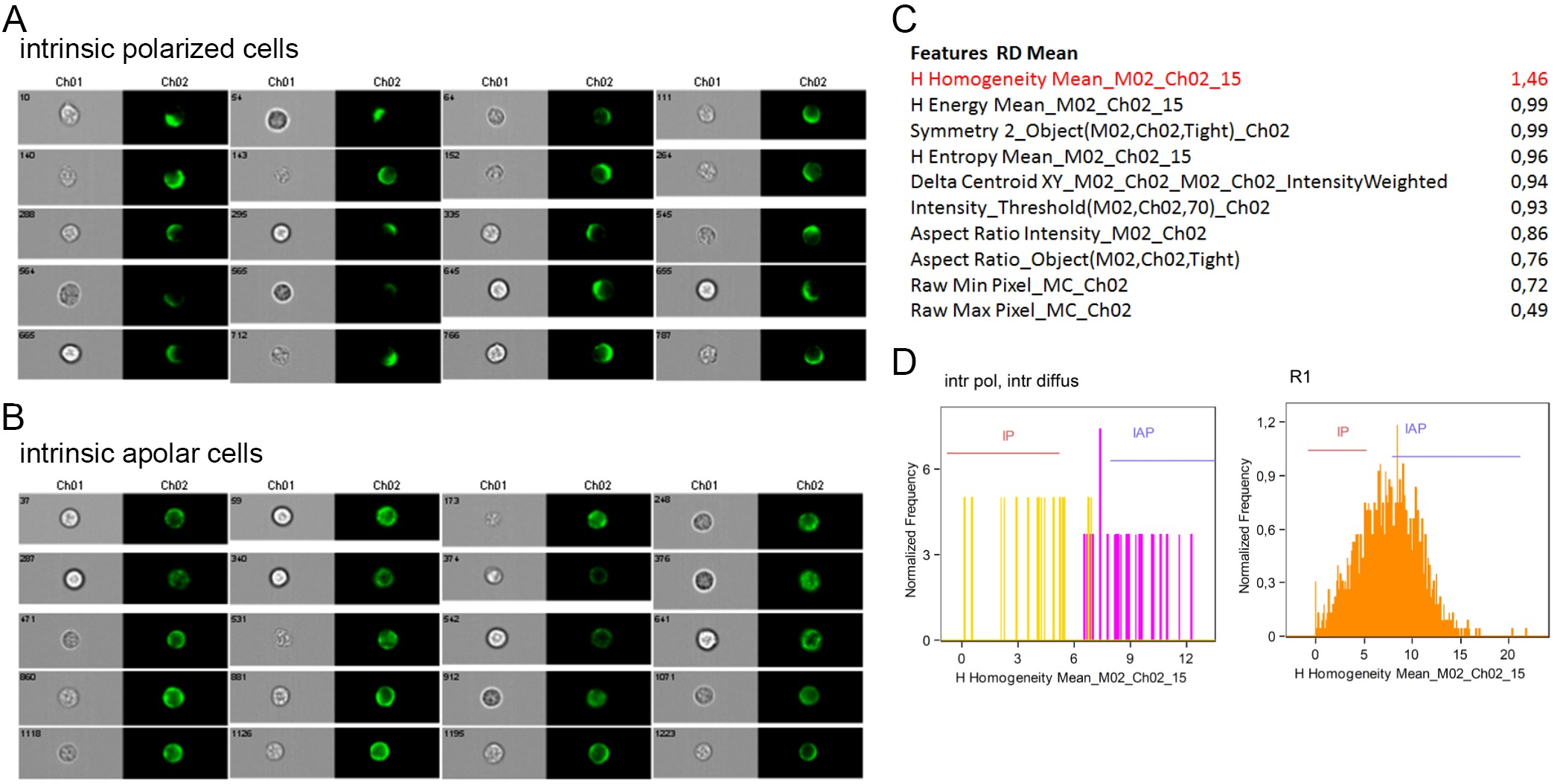

Supplement: S1 Fig — CLL cells were analyzed by IFCM as described in Fig 2. For morphologically round cells with polar (A) and diffuse (B) distribution of CD50 the Homogeneity Feature combined with the channel mask of channel 2 (M02) was applied to discriminate both cell populations in quantitative manners (C, D). (TIF) [file pone.0228674.s001.tif]

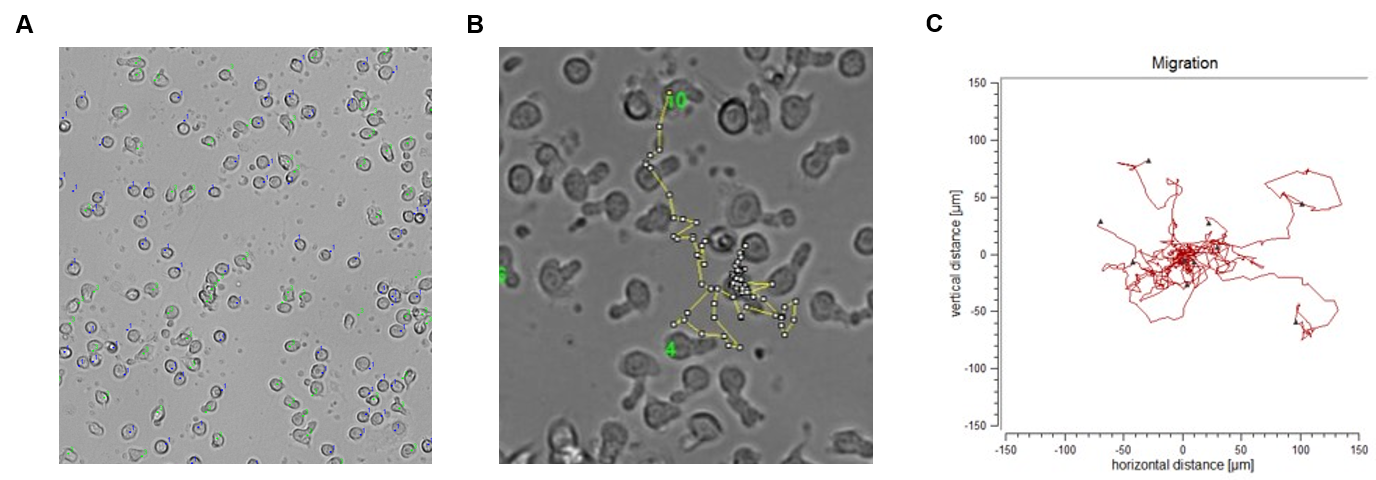

Supplement: S2 Fig — A: The fraction of polarized cells was determined by manual counting in ImageJ Green markers in the given example represent morphologically polarized cells, blue markers represent unpolarized cells. B: One single cell was tracked on 100 pictures of time-lapse microscopy and the track was analyzed by a Manual tracking plugin for ImageJ. C: Migration tracks of 10 polarized cells depicted by Chemotaxis and Migration tool. (TIF) [file pone.0228674.s002.tif]

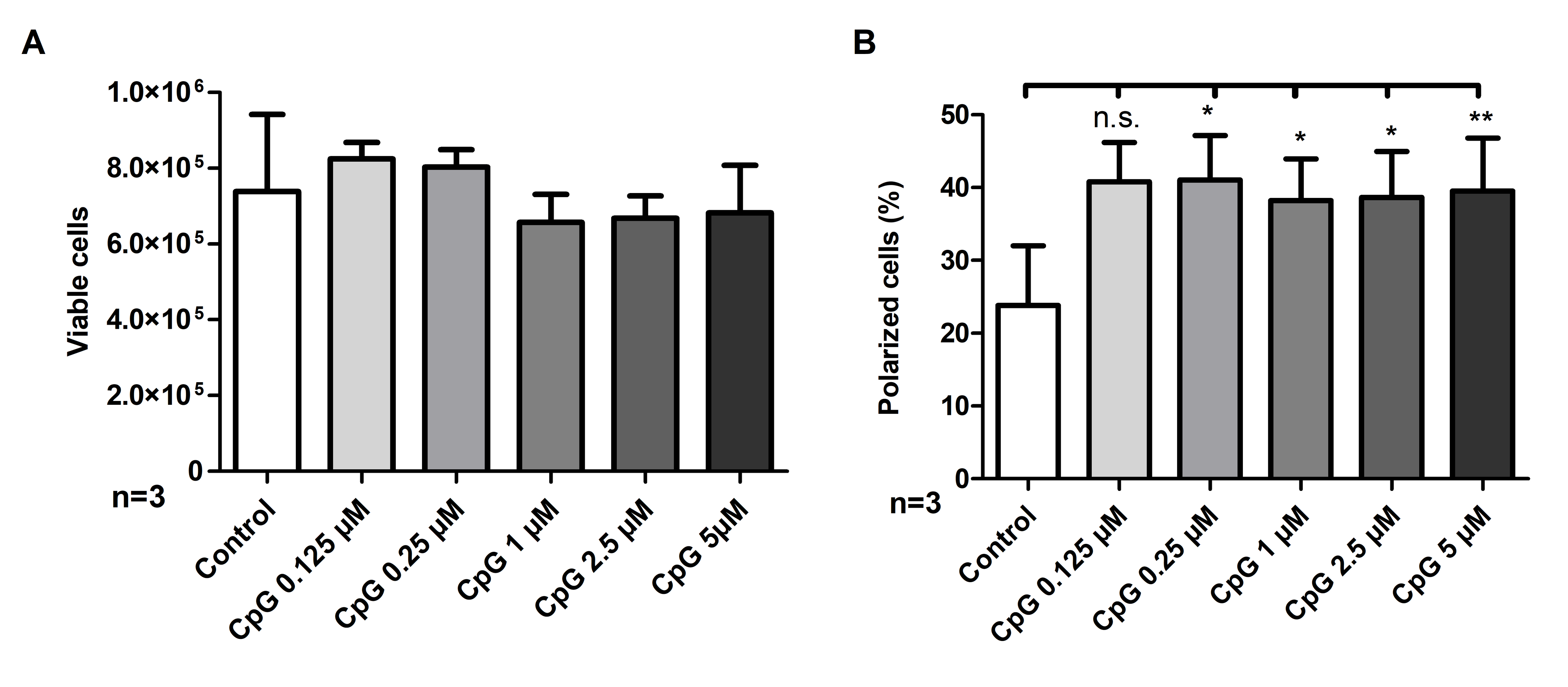

Supplement: S3 Fig — CLL cells were incubated with rising concentration of CpG type B. Cell viability (A) and cell polarization (B) were assessed after 48h hours. (TIF) [file pone.0228674.s003.tif]
