# Supplementary material for: CpG stimulation of chronic lymphocytic leukemia cells induces a polarized cell shape and promotes migration in vitro and in vivo
Source: PLoS One. 2020 Feb 10;15(2):e0228674. doi: 10.1371/journal.pone.0228674 (PMC7010256; doi:10.1371/journal.pone.0228674)
Supplement: S1 Table — (DOCX) [file pone.0228674.s005.docx]

|  | Cytogenetics | Thymidine Kinase U/l | CD38 / ZAP 70 | IgVH mutation status | WBC at sampling /nl | Spontaneus polarization (%) |
| --- | --- | --- | --- | --- | --- | --- |
| Patient 1 | del 13q14 | Unknown | Positive / Positive | Unknown | 64 | 10.1 |
| Patient 2 | Unknown | 6.9 | Negative / Unknown | Mutated | 149 | 30.4 |
| Patient 3 | del 11q, del 13q14 | 3.9 | Negative / Positive | Unmutated | 50 | 38.8 |
| Patient 4 | Del11q, del 13q14 | 17.1 | Positive / Unknown | Unknown | 70 | 16.3 |
| Patient 5 | Del13q14 | 10.1 | Negative / Unknown | Unknown | 33 | 28.4 |
| Patient 6 | Del13q14 | 4.1 | Negative / Unknown | Unknown | \| 98 \| \| --- \| | 10.7 |
| Patient 7 | Unknown | 5.3 | Negative/ Negative | Unknown | 49 | 34.7 |
| Patient 8 | Del13q14 | 4.9 | Negative / Positive | Unknown | 47 | 18.7 |
